# Outcome of children with multiply relapsed B-cell acute lymphoblastic leukemia: a therapeutic advances in childhood leukemia & lymphoma study

**DOI:** 10.1038/s41375-018-0094-0

**Published:** 2018-03-15

**Authors:** Weili Sun, Jemily Malvar, Richard Sposto, Anupam Verma, Jennifer J. Wilkes, Robyn Dennis, Kenneth Heym, Theodore W. Laetsch, Melissa Widener, Susan R Rheingold, Javier Oesterheld, Nobuko Hijiya, Maria Luisa Sulis, Van Huynh, Andrew E. Place, Henrique Bittencourt, Raymond Hutchinson, Yoav Messinger, Bill Chang, Yousif Matloub, David S. Ziegler, Rebecca Gardner, Todd Cooper, Francesco Ceppi, Michelle Hermiston, Luciano Dalla-Pozza, Kirk R. Schultz, Paul Gaynon, Alan S. Wayne, James A. Whitlock

**Affiliations:** 1 City of Hope National Medical Center, Duarte, CA USA; 2 Children’s Center for Cancer and Blood Diseases, Children’s Hospital Los Angeles, Los Angeles, CA USA; 3 Children’s Center for Cancer and Blood Diseases, Children’s Hospital Los Angeles, USC Norris Comprehensive Cancer Center, Keck School of Medicine, University of Southern California, Los Angeles, CA USA; 4 Primary Children’s Hospital, Pediatric Hematology/Oncology, University of Utah, Salt Lake City, UT USA; 5 Seattle Children’s Hospital, University of Washington, Seattle, WA USA; 6 Nationwide Children’s Hospital, the Ohio State University, Columbus, OH USA; 7 Cook Children’s Medical Center, Fort Worth, TX USA; 8 UT Southwestern Medical Center/ Children’s Health, Dallas, TX USA; 9 Center for Cancer & Blood Disorders, Children’s Hospital Colorado, Aurora, CO USA; 10 Children’s Hospital of Philadelphia, Philadelphia, PA USA; 11 Levine Children’s Hospital at Carolinas Medical Center, Charlotte, NC USA; 12 Ann & Robert H Lurie Children’s Hospital of Chicago, Northwestern University Feinberg School of Medicine, Chicago, IL USA; 13 New York Presbyterian Hospital, Columbia University Medical Center, New York, NY USA; 14 Children’s Hospital Orange County, Hyundai Cancer Institute, Orange, CA USA; 15 Dana-Farber/Boston Children’s Cancer and Blood Disorders Center, Harvard Medical School, Boston, MA USA; 16 CHU Sainte-Justine, Universite de Montreal, Montreal, QC Canada; 17 C.S Mott Children’s Hospital, University of Michigan, Ann Arbor, MI USA; 18 Children’s Hospital of Minnesota, Minneapolis, MN USA; 19 Doernbecher Children’s Hospital, Knight Cancer Institute, Oregon Health & Science University, Portland, OR USA; 20 Rainbow Babies and Children’s Hospital, Case Western Reserve University School of Medicine, Cleveland, OH USA; 21 Kids Cancer Center, Sydney Children’s Hospital, School of Women’s and Children’s Health, University of New South Wales, Sydney, NSW Australia; 22 Department of Pediatrics, University of Lausanne, Lausanne, Switzerland; 23 Benioff Children’s Hospital, University of California, San Francisco, CA USA; 24 Cancer Center for Children, The Children’s Hospital at Westmead, Westmead, NSW Australia; 25 BC Children’s Hospital and Research Institute, University of British Columbia, Vancouver, BC Canada; 26 The Hospital for Sick Children, University of Toronto, Toronto, ON Canada

## Abstract

The survival of pediatric patients with multiply relapsed and/or refractory (R/R) B-cell acute lymphoblastic leukemia has historically been very poor; however, data are limited in the current era. We conducted a retrospective study to determine the outcome of multiply R/R childhood B-ALL treated at 24 TACL institutions between 2005 and 2013. Patient information, treatment, and response were collected. Prognostic factors influencing the complete remission (CR) rate and event-free survival (EFS) were analyzed. The analytic set included 578 salvage treatment attempts among 325 patients. CR rates (mean ± SE) were 51 ± 4% for patients with bone marrow R/R B-ALL who underwent a second salvage attempt, 37 ± 6% for a third attempt, and 31 ± 6% for the fourth through eighth attempts combined. For patients achieving a CR after their second, third, and fourth through eighth attempts, the 2 year EFS was 41 ± 6%, 13 ± 7%, and 27 ± 13% respectively. Our results showed slight improvement when compared to previous studies. This is the largest and most recent study to date that evaluates the outcome of this patient population. Our data will provide detailed reference for the evaluation of new agents being developed for childhood B-ALL.

## Introduction

Acute lymphoblastic leukemia (ALL) is the most common malignancy in children and adolescents, with ~85% of cases being B-cell precursor ALL (B-ALL). Over the past few decades, the overall survival rate in children with newly diagnosed ALL has improved dramatically from ~10% in the 1960s to almost 90% today [1, 2]. Despite this remarkable improvement, ~2% patients are refractory to induction chemotherapy [3], and an additional 10–15% of ALL patients still experience a relapse [4]. Although subsequent second complete remission (CR) can be achieved in most patients [5–8], ~55% of those patients will relapse again [6, 9]. Those children are generally managed with intensive chemotherapy, with or without novel agents to induce a third remission, followed by hematopoietic stem cell transplant (HSCT) if indicated [10, 11]. Despite the improvement of outcome in newly diagnosed patients, the reported event-free survival (EFS) of patients with first relapse of ALL has not changed significantly for more than 20 years and remains poor at ~35–50% [5–9, 12, 13]. The outcome for patients who fail initial induction therapy (primary induction failure), for those who do not respond to salvage therapy, and for those who are multiply relapsed is even worse. Therefore, new strategies are needed to improve the outcome of these patients.

The Therapeutic Advances in Childhood Leukemia consortium (TACL) was established in 2004 to develop innovative therapies through phase I/II clinical trials in children with incurable leukemia and lymphoma. Previously, TACL conducted a retrospective study to evaluate the remission rates and outcomes for children with refractory or multiply relapsed (R/R) ALL treated at eight TACL institutions in the United States (US) from 1995 to 2004 [5]. A CR rate of approximate 40% was identified in children who experienced second and subsequent relapse. Several other studies reported similar outcomes in children with multiply R/R ALL [10, 11]. The data provided reference information for clinicians and families to make treatment decisions and serve as a benchmark for the evaluation of new agents and regimens [5]. However, these studies may not reflect the current practice as treatment patterns and supportive care measures have changed over the past 10 years.

To provide current and precise estimates of outcome in children with multiply R/R B-ALL, we performed a more comprehensive follow-up study using pooled retrospective data collected from 24 TACL institutions in the US, Canada, and Australia. The primary objective of the study was to estimate the CR rate in pediatric patients with multiply R/R or primary induction failure B-ALL treated according to the institutional standard of care at participating centers. The secondary objectives were to estimate the EFS probabilities in this patient population, and to investigate patient and disease characteristics that are associated with these primary and secondary objectives.

## Subjects and methods

### **P**atients

The TACL T2014–004 study included patients ≤21 years with R/R B-ALL who experienced a qualifying treatment failure at a TACL institution between 2005 and 2013. Qualifying treatment failures included patients who underwent salvage treatment for primary induction failure, or with ≥2 occasions of relapsed disease; or failure to achieve remission after first or more salvage treatment attempt.

Patients meeting the eligibility criteria for this study were identified at each participating TACL institution. The approach for identifying potentially eligible patients included: tumor registries, medical records, hospital billing records, and internally maintained patient databases to ensure a complete census of eligible patients. Patient demographic information and clinical data related to the initial diagnosis and subsequent treatment failures were abstracted from the medical record. Collected data included disease characteristics, chemotherapy regimen, disease response, and survival until the date of death or end of follow up at least through 31 December 2014. Data were entered in the TACL DataLabs Clinical Data Management System and reviewed centrally. This study was approved by the institutional review board of each participating institution.

### Definitions

A salvage treatment attempt was defined as a chemotherapy treatment plan initiated because of a relapsed or refractory leukemia. A curative attempt was defined as a treatment plan with the goal of achieving a CR. To determine whether a chemotherapy plan was curative or palliative, therapy regimens were evaluated and classified by two independent reviewers. A third reviewer participated when the two reviewers did not agree. Palliative attempts were excluded for all analyses that used response as a dependent variable. The outcomes of these palliative attempts were classified as “not evaluable” in analyses using prior treatment response as an independent variable predictive of subsequent response.

Response was evaluated using the complete blood count, bone marrow (BM), and extramedullary disease evaluations collected at the end of each treatment. Patients were considered to have achieved a CR if there were ≤5% blasts in the BM and no evidence of extramedullary disease. Relapse referred to leukemia recurrence in the BM, central nervous system (CNS), or other extramedullary sites following a CR. CNS leukemia was defined as CNS3 disease (≥5/µl WBCs and positive for blasts, or clinical signs of CNS leukemia). Medullary relapse was defined as >5% blasts in the BM. Isolated extramedullary relapse was defined as ≤5% blasts in the BM and evidence of disease in CNS, testicular, or other extramedullary sites. Combined relapse was defined as >5% blasts in the BM and evidence of extramedullary leukemia. Refractory disease was defined as failure to achieve CR after one course of curative chemotherapy. Primary induction failure was defined as failure to achieve CR after one course of induction chemotherapy for de novo ALL. Patients who had peripheral blasts ≥25% by morphology without BM assessments were designated to have relapse or refractory disease. Induction death was defined as death within 30 days from the initiation of systemic salvage chemotherapy.

The duration of prior remission in patients achieving CR was defined as the time between relapse date and the date of the previous CR. EFS was measured from the time of remission confirmation, to the date of relapse or death from any cause, or was censored at the earliest of the date of last follow up or 31 December 2014.

### Statistical methods

Response and EFS analyses used salvage attempt as the unit of analysis. Attempts were excluded from these analyses if data were insufficient to determine outcome, if the treatment was judged as palliative, or if the attempt was prior to the patient being first seen at the TACL institution. The latter exclusion was to eliminate selection bias of treatment attempts that were more likely to be successful, as patients with unsuccessful treatment attempts (e.g., resulting in death) would be less likely to present at a TACL institution.

Univariable and multivariable logistic regression was used to analyze reinduction failure rates at the first and later salvage attempts. Independent variables included salvage treatment attempt number, prior remission duration, National Cancer Institute (NCI) risk criteria [14] at time of initial diagnosis, extramedullary and BM status at the start of the treatment attempt, and cytogenetics at diagnosis.

Cox regression analysis was used to examine the influence of these independent variables on EFS following CR. These analyses were restricted to salvage treatment attempts where patients achieved remission at their second or later salvage attempt. The analysis of CR rate and EFS used salvage attempts rather than patients as the primary analytic unit, so that each patient contributed data on one or more attempts. As in our previous publication [5] the corresponding logistic and Cox regression analyses, accounting for this inter-patient correlation, gave equivalent results to analyses that ignored this correlation. Results from the latter analytic method are reported. The administration of HSCT after salvage was included as a time-dependent covariate in the Cox regression analysis.

All *p*-values are two-sided tests, and estimates of relative risk and relative failure rate are presented with 95% confidence intervals. Statistical computation was performed using Stata 11 (StataCorp. 2009. Stata Statistical Software: Release 11. College Station, TX: StataCorp LP).

## Results

### Analysis cohort

**Fig. 1:**
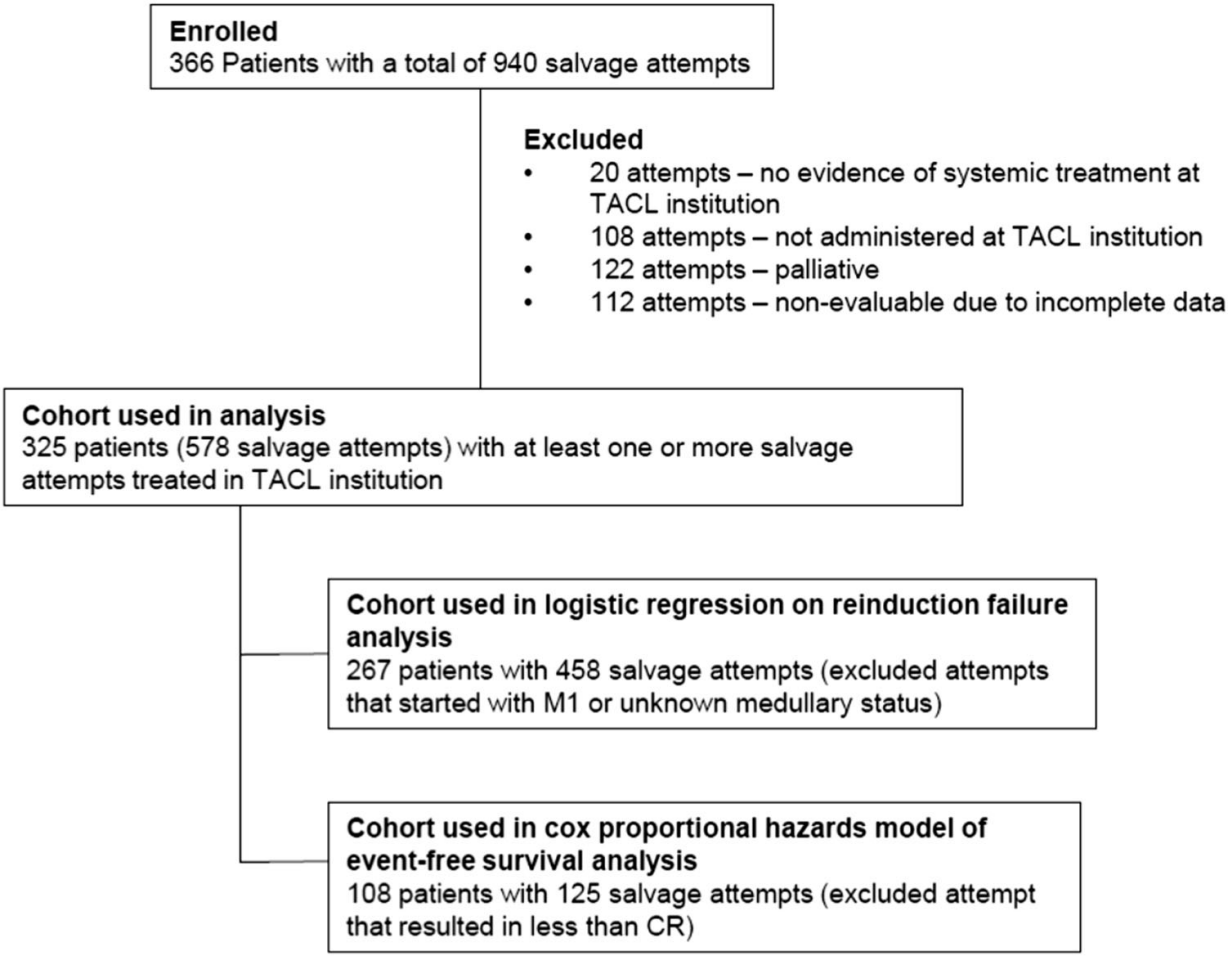
Consort diagram

A total of 366 unique patients from 24 TACL institutions with a total of 940 salvage attempts were enrolled in this study. The analytic set included 578 first and greater salvage treatment attempts among 325 patients (Fig. 1). Reasons for exclusion included: no evidence of systemic treatment (*n* = 20 attempts); attempts that were administered prior to the patient’s treatment at a TACL institution (*n* = 108 attempts); attempts that were determined as palliative (*n* = 122 attempts); and attempts that were not evaluable for response due to incomplete or missing data (*n* = 112 attempts). Those attempts were not included in the analytic set, although their information could be used as independent variables in analysis. The clinical characteristics of the patients at initial diagnosis are summarized in Table 1.

**Table 1:** Characteristics at initial diagnosis of patients with ALL who received at least one salvage attempt (*n* = 325)

Characteristic	Levels	Number of patients	%
Age, years	<1 (infants)	28	8.6
	1–9	183	56.3
	10 and over	113	34.8
	Unknown	1	0.3
WBC counts/µL	<50,000/µl	189	58.1
	50,000/µl and over	89	27.4
	Unknown	47	14.5
NCI risk criteria at diagnosis	Non-infants, standard risk	114	35.1
	Non-infants, high risk	137	42.1
	Non-infants, unknown	46	14.2
	Infants	28	8.6
Sex	Female	133	40.9
	Male	192	59.1
Testicular disease	Yes	3	0.9
	No	189	58.2
	N/A	133	40.9
CNS leukemia (CNS3)	Yes	69	21.2
	No	225	69.3
	Unknown	31	9.5
*Cytogenetics* ^a^			
Favorable	t(12;21)	10	3.1
	Hyperdiploidy (>50 chromosomes)	41	12.6
Unfavorable	11q23 (KMT2A gene) rearranged	39	12.0
	Hypodiploidy (<45 chromosomes)	14	4.3
	iAMP21	3	0.9
	t(9;22)	20	6.2
Other	Normal	103	31.7
	t(1;19)	9	2.8
	9p abnormality	10	3.1
	Other	32	9.8
	Unknown	44	13.5

*CNS* central nervous system, *NCI* National Cancer Institute

^a^ Main Karyotype presented here; 23 patients had multiple entries

The majority of salvage attempts were due to BM relapse (458/578, 79.2%), while 13.5% were due to isolated extramedullary disease (Supplementary Table S1). BM status was unclear for the remaining 7.3% of the salvage attempts for extramedullary disease (Supplementary Table S1). Due to the complexity of the treatment, the salvage attempts were grouped into chemotherapy only, attempts with chemotherapy with a novel agent, and attempts with chemotherapy with HSCT (Supplementary Table S2). Fifty-eight salvage attempts (10%) included novel agent. HSCT was included in 31% salvage attempts.

### Response to salvage treatment attempts

Since the majority of multiply R/R disease occurred in the BM, we focused our analysis of the response rate for treatment of two and more BM (isolated and combined) relapses. This comprised 267 unique patients with 458 salvage treatment attempts (Fig. 1). Table 2 summarizes the number of salvage attempts resulting in CR by whether or not a previous remission was achieved and the length of the previous remission at the specified salvage treatment attempt. The overall CR rate was 69 ± 3.6% after the first salvage treatment attempt, 51 ± 3.9% after the second salvage attempt, and <40% after the third and subsequent attempts (Table 2). Among the 25 patients with primary induction failure, 13 patients (52%) achieved CR after first salvage treatment attempt (Table 2). There were 16 induction deaths among the 458 curative salvage attempts (3.5%).

**Table 2:** Achievement of CR after treatment of bone marrow disease at reporting TACL institutions (*n* = 267 unique patients with 458 salvage attempts)

	First salvage attempt			Second salvage attempt			Third salvage attempt			Fourth through eighth salvage attempt		
Duration of previous CR	Total	CR	%	Total	CR	%	Total	CR	%	Total	CR	%
CR not achieved	25	13	52	43	17	40	35	11	31	30	9	30
CR < 18 months duration	45	25	55	52	22	42	17	6	35	5	2	40
CR 18–36 months duration	40	32	80	26	18	69	2	1	50	3	1	33
CR > 36 months duration	37	30	81	16	15	94	2	2	100	–	–	–
Prior CR not evaluable	21	16	76	28	12	43	17	7	41	14	4	29
All patients combined	168	116	69	165	84	51	73	27	37	52	16	31

*CR* complete remission

The results of the logistic regression for reinduction failure occurring at the first and later salvage attempts are displayed in Table 3. Salvage attempt number, duration of previous remission, and NCI risk category at diagnosis were all significant predictors in both univariable and multivariable analyses (Table 3). In the multivariable model, increasing salvage attempt number was associated with increased risk of reinduction failure (trend *p* = 0.0001). Duration of prior remission was inversely correlated with risk of reinduction failure (trend *p* = 0.0028). Patients with a high or unknown NCI risk category at initial diagnosis or infant ALL were also associated with higher risk of reinduction failure compared to patients classified as NCI standard risk (*p* = 0.0322). Neither extramedullary involvement nor BM status (M2 vs. M3) at start of therapy was associated with reinduction failure in univariable and multivariable analyses. Unfavorable cytogenetics at diagnosis was associated with higher risk of reinduction failure compared to patients having favorable and other cytogenetics in univariable analysis (*p* = 0.0429), but not in multivariable analysis (*p* = 0.7991).

**Table 3:** Summary of logistic regression for reinduction failure for medullary disease at reporting TACL institutions (267 unique patients with 458 salvage attempts)

		Response		Univariable analysis		Multivariable analysis	
Variable	Variable levels	CR	Failure	OR	95% CI	OR	95% CI
Salvage attempt	1	116	52	1.0	–	1.0	–
	2	84	81	2.15	1.37–3.36	1.83	1.12–2.99
	3 and over	43	82	4.25	2.60–6.96	3.02	1.72–5.31
	*p*-value			**<0.0001**		**0.0004**	
	Trend *p*-value			**<0.0001**		**0.0001**	
Duration of previous remission (CR)	CR not achieved	48	80	1.39	0.84–2.31	1.20	0.69–2.08
	CR achieved, < 18 m duration	56	67	1.0	–	1.0	–
	CR achieved, 18–36 m duration	52	19	0.31	0.16–0.56	0.37	0.19–0.71
	CR achieved, > 36 m duration	48	8	0.14	0.06–0.32	0.19	0.08–0.46
	Not evaluable for response	39	41	0.88	0.50–1.54	0.70	0.37–1.29
	*p*-value			**<0.0001**		**<0.0001**	
	Trend *p*-value			**0.0021**		**0.0028**	
NCI risk category at diagnosis	Non-infants, standard risk	110	63	1.0	–	1.0	–
	Non-infants, high risk	100	103	1.80	1.19–2.72	1.62	1.03–2.56
	Non-infants, unknown	23	31	2.35	1.26–4.38	1.80	0.90–3.60
	Infants	10	18	3.14	1.37–7.23	3.06	1.25–7.51
	*p*-value			**0.0020**		**0.0322**	
Extramedullary involvement at start of therapy	No	194	171	1.0	–	1.0	–
	Yes	49	44	1.02	0.64–1.61	1.21	0.73–2.01
	*p*-value			0.9364		0.4499	
BM status at start of therapy	M2	39	35	1.0	–	1.0	–
	M3	204	180	0.98	0.59–1.62	1.45	0.83–2.54
	*p*-value			0.9469		0.1916	
Cytogenetics	Favorable	49	29	1.0	–	1.0	–
	Unfavorable	47	59	2.12	1.17–3.86	1.2	0.59–2.45
	Other	147	127	1.46	0.87–2.45	1.0	0.55–1.83
	*p*-value			**0.0429**		0.7991	

*BM* bone marrow, *CI* confidence interval, *CR* complete remission, *NCI* National Cancer Institute, *OR* odds ratio. The bold values are the p-values with statstical significance (< 0.05)

Comparing the unadjusted CR rate among patients who received ≥2 salvage attempts between our study and the previous TACL study, we identified an improved CR rate for patients who received fourth through eighth attempts, in whom 31% of patients achieved CR (Table 4) vs. 12% in the prior study (*p* = 0.014) [5].

**Table 4:** Comparison of unadjusted CR rates of patients with medullary relapsed/refractory ALL between two sequential TACL studies

Number of salvage attempt	CR rate (SE) [95% confidence interval]		Difference (Sun–Ko) (SE) (testing proportion)
	1995–2004 (Ko et al.) [5]	2005–2013 (Sun et al.)	
Second salvage attempt	44.44 % (4.78) [34.88, 54.32]	50.91 % (3.89) [43.02, 58.76]	0.0647 (0.0616) (−0.0561, 0.1855) * p* = 0.2955
Third salvage attempt	26.78 % (5.92) [15.83, 40.30]	36.99 % (5.65) [25.97, 49.09]	0.1021 (0.0818) (−0.0583, 0.2624) * p* = 0.2200
Fourth through eighth salvage attempt	12.31 % (4.07) [5.47, 22.82]	30.77 % (6.40) [18.72, 45.10]	0.1846 (0.0759) (0.0358, 0.3333) * p* = 0.0140

*CR* complete remission, *SE* standard error

### 2 year EFS for patients achieving CR

**Fig. 2:**
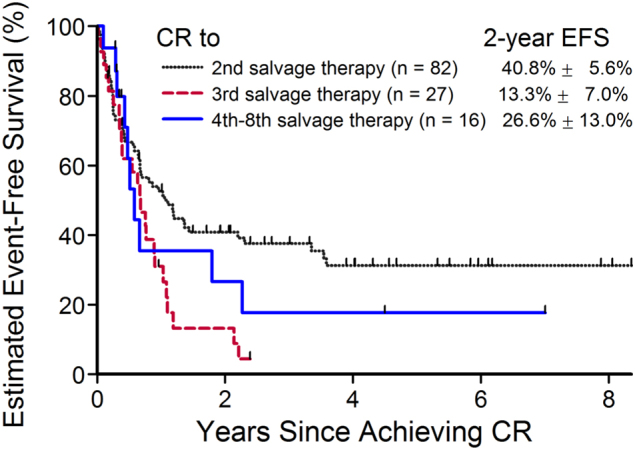
Estimated 2 year event-free survival for patients who achieved complete remission after ≥2nd salvage attempt. CR complete remission, EFS event-free survival

Next, we focused on factors that impacted 2 year EFS among patients with at least two salvage attempts who subsequently achieved a CR. A total of 286 patients were identified who met these criteria. Among patients undergoing ≥2 salvage attempts, 125 attempts (in 108 unique patients) resulted in CR (Fig. 1). Survival analysis was completed on this cohort of patients. Kaplan–Meier survival curves by treatment attempts are provided in Fig. 2.

To investigate the prognostic factors that can impact the survival after achieving CR, Cox regression univariable and multivariable analyses were performed and presented in Table 5. Prior number of salvage attempts was the only significant predictor in the univariable analysis of EFS time among patients who achieved CR after a second or greater salvage attempt (*p* = 0.0323, trend *p* = 0.0906). However, in the multivariable analysis, the effect of the salvage attempt number is attenuated. Duration of previous remission, NCI risk category, extramedullary involvement, HSCT post-remission, BM status, and cytogenetics at start of therapy all failed to reach statistical significance in both univariable and multivariable analysis. In addition, in the multivariable analysis, a trend of decreasing risk of relapse/death among patients with longer duration of prior remission for patients with 18–36 months and at least 36 months duration of previous remission was seen compared to patients that achieved a prior remission lasting less than 18 months.

**Table 5:** Cox proportional hazards model of event-free survival from start of remission for patients who achieved CR after ≥2 salvage attempts (*n* = 108 patients with 125 attempts)

Variable	Variable levels	Number of events/total	Univariable analysis		Multivariable analysis	
			HR	95% CI	HR	95% CI
Salvage attempt	Second	52/82	1.0	–	1.0	–
	Third	24/27	1.99	1.21–3.26	1.78	1.06–2.98
	Fourth through eighth	10/16	1.31	0.67–2.60	1.18	0.57–2.44
	*p*-value		**0.0323**		0.1073	
	Trend *p*-value		0.0906		0.1368	
Duration of previous remission	CR not achieved	26/37	0.72	0.40–1.28	0.77	0.40–1.47
	CR < 18 m duration	21/29	1.0	–	1.0	–
	CR 18–36 m duration	12/20	0.61	0.30–1.25	0.71	0.34–1.49
	CR ≥ 36 m duration	8/16	0.41	0.18–0.92	0.45	0.19–1.04
	Not evaluable for response	19/23	1.16	0.62–2.15	1.20	0.61–2.36
	*p*-value		0.1452		0.2807	
	Trend *p*-value		0.1422		0.2211	
NCI risk category at diagnosis	Non-infants, standard risk	39/60	1.0	–	1.0	–
	Non-infants, high risk	35/46	1.29	0.81–2.02	1.21	0.74–1.97
	Non-infants, unknown	11/17	1.08	0.55–2.11	0.96	0.44–2.11
	Infants	1/2	–	–	–	–
	*p*-value		0.7440		0.8127	
Extramedullary involvement at relapse	No	72/101	1.0	–	1.0	–
	Yes	14/24	0.67	0.38–1.19	0.71	0.38–1.31
	*p*-value		0.1522		0.2644	
Subsequent HSCT	No	43/57	1.0	–	1.0	–
	Yes	43/68	0.73	0.45–1.21	0.76	0.42–1.36
	*p*-value		0.2292		0.3530	
BM status at start of attempt	M2 (5–25%)	18/23	1.0	–	1.0	–
	M3 (over 25%)	68/102	0.90	0.53–1.51	1.14	0.65–1.99
	*p*-value		0.6854		0.6500	
Cytogenetics	Favorable	17/24	1.0	–	1.0	–
	Unfavorable	14/21	1.02	0.50–2.07	0.53	0.22–1.28
	Other	55/80	1.08	0.68–2.03	1.15	0.63–2.10
	*p*-value		0.7802		0.0869	

*BM* bone marrow, *CR* complete remission, *HR* hazard ratio, *HSCT* hematopoietic stem cell transplant. The bold values are the p-values with statstical significance (< 0.05)

Although minimal residual disease (MRD) data were available for some salvage attempts (40 attempts), most of the patients did not have MRD data available. Therefore, MRD was excluded in the analysis.

## Discussion

This is the second retrospective pooled data analysis from TACL that evaluates the outcome of pediatric patients with multiply R/R B-ALL treated during a contemporary period. Efforts were made to include a complete census of eligible patients from each participating center in order to minimize patient selection bias. Twenty-four TACL institutions participated in the study, representing major pediatric hematology/oncology centers across the US, Canada, and Australia. The inclusion of 325 patients provided us with the opportunity to undertake a robust analysis and evaluate factors that influenced the remission rate and survival.

Comparison of the current study with the previous TACL study is not straight forward, since the majority of our patients had ≥2 occasions of relapses, whereas the previous study also included patients with first relapsed disease [5]. Therefore, not surprisingly, the CR rate among our cohort after a 2nd treatment attempt is biased as it excludes patients who had a single treatment failure, achieved remission, and remained in remission, and slightly less than the reported CR rate of 81–94% in the literature [5–8]. We observed a trend of improved response rate for patients who had two or more salvage attempts in our study when compared to the results reported by Ko et al. and other published studies [5, 10, 11]. More importantly, we identified a significantly higher CR rate among patients who received fourth through eighth salvage attempts in the current study when compared to the previous TACL study. Considering the improvement of the treatment of *de novo* ALL in recent time periods, one might assume that remission would be harder to achieve in these multiply salvaged patients. However, our finding is consistent with the previous report from the Children’s Oncology Group suggesting that post-relapse survival is independent of initial treatment intensity in children with first relapsed ALL [9]. This observation is also consistent with the findings of several genomic studies that indicate that clones responsible for relapse are often present at diagnosis or mutated to a resistant phenotype through intrinsic genomic instability rather than treatment exposures [15, 16]. We speculate this apparent improvement in CR rate could be related to intensification of salvage chemotherapy, introduction of novel agents in this patient population in recent time periods, and better supportive care. However, the difference could also be attributed to differences in non-treatment-related features of patients in these two groups.

As previously published, the number of prior salvage attempts and duration of previous remission are the prognostic factors contributing to the subsequent CR in children with multiply relapsed ALL [5, 10, 11]. We found no compelling association of reinduction failure with either extramedullary involvement or BM status (M2 vs. M3) at start of therapy in univariable or multivariable analyses. Our study identified that NCI risk category at diagnosis is a significant independent prognostic factor for remission induction. This observation was consistent with a non-significant trend observed in the previous TACL publication [5]. However, after achieving CR, it had no impact in the survival of these patients. Although several studies have suggested that cytogenetics at diagnosis was an independent prognostic factor in children with ALL in first relapsed or primary induction failure [3, 13], the impact of cytogenetics in children with multiply relapsed ALL was unknown. In our study, unfavorable cytogenetics was associated with higher risk of induction failure only in univariable analysis. There appears to be a trend of lower disease progression among patients with unfavorable cytogenetics when CR was achieved after ≥2 salvage attempts in multivariable analysis in our small cohort. Given the wide 95% confidence interval, further studies is warranted. Taken together, these data highlight the importance of understanding the biology in relapsed ALL to identify targets for novel therapies that can result in more sustained CR.

Few studies have evaluated survival in patients who achieved CR3 and beyond. Previous studies reported a 23–31% EFS in patients achieving CR3 [4, 11, 12]. In our analysis, we found an improved 2 year EFS for patients who achieved a CR3 (41% ± 5.6%). With small patient numbers, we found no compelling advantage for HSCT in patients who achieved CR after ≥2 salvage attempts. Furthermore, a 2 year EFS of 27 ± 13% was seen in patients who had ≥4 salvage attempts, a slight improvement from the previous TACL study, although the number of patients are very small. Of note, a total of 61 salvage attempts were administered to 16 patients as their fourth eighth attempts. Among the 61 salvage attempts, only five (8%) were CD19 chimeric antigen receptor (CAR) T cell therapy. Therefore, the improved CR rate among this group could not be solely explained by the highly effective CD19 CAR T cell immunotherapy. However, it is possible this treatment could have resulted in deeper and more sustained CR and have influenced the EFS in these 16 patients who experienced five or more prior treatment failures.

Overall, compared to previous studies, our data demonstrated that in the contemporary era, more effective reinduction therapy resulted in a trend of higher CR rates, more sustained remissions and improved survival. Despite these improvements, the majority of patients in our cohort died from their disease. Therefore, new approaches are still needed to improve outcomes. The results from our data provide important reference background information for evaluating CR rates in future early phase clinical trial designs for B-ALL, especially with respect to the composition of patient characteristics in those new agent trials which typically included multiply relapsed/refractory patients. However, it will always be important to consider the limitations of historical data when using them as reference in clinical trials because retrospective clinical data are not equivalent to clinical trial data. Other limitations of our analysis included, the lack of data regarding organ function, performance status, other co-morbidities, and enrollment in investigational studies. Treatment-related adverse events and therapy modifications due to toxicity were not collected. Only a few patients had MRD data available; therefore, the impact of MRD in the outcome is unknown. Out of the 325 patients, only a small number of patients received CD19 or CD22 directed immunotherapy (blinatumomab, *n* = 7; CD19 CAR T cell, *n* = 11; inotuzumab, *n* = 1) [17–19]. Therefore, our analysis reflects the treatment outcome prior to the CD19 directed immunotherapy era. It will be interesting to see whether the introduction of new promising agents such as blinatumomab, inotuzumab, and CD19 CAR T cell therapy will change the long-term outcome in these patients.

In conclusion, this is the largest retrospective study to date of the outcome of children with multiply R/R B-ALL receiving contemporary treatment across North America and Australia, and demonstrated a trend of improvement in CR rate and survival compared to the previous TACL study. The pooled data provide important background information in the outcome of children with multiply R/R B-ALL, which can be valuable in planning clinical trials assessing new drugs and biologic agents.

## Electronic supplementary material

Supplemental data(DOCX 14 kb)
