# Supplementary material for: Outcome of children with multiply relapsed B-cell acute lymphoblastic leukemia: a therapeutic advances in childhood leukemia & lymphoma study
Source: Leukemia. 2018 Mar 15;32(11):2316–25. doi: 10.1038/s41375-018-0094-0 (PMC6224404; doi:10.1038/s41375-018-0094-0)
Supplement: Supplementary file 1 — Supplemental data(DOCX 14 kb) [file 41375_2018_94_MOESM1_ESM.docx]

**Supplementary Table S1. Medullary or extra medullary relapse/failure at start of salvage attempt**

|  |  | **Types of Relapse/Failure** | | |
| --- | --- | --- | --- | --- |
| **Salvage Attempt** | **Number of Patients Treated** | **Medullary** | **Isolated**  **Extra-medullary** | **Extramedullary/**  **Unknown medullary** |
| **First** | 221 | 168 | 46 | 7 |
| **Second** | 201 | 165 | 23 | 13 |
| **Third** | 89 | 73 | 6 | 10 |
| **Fourth** | 31 | 26 | 1 | 4 |
| **Fifth** | 21 | 15 | 2 | 4 |
| **Sixth** | 11 | 8 | 0 | 3 |
| **Seventh** | 3 | 2 | 0 | 1 |
| **Eighth** | 1 | 1 | 0 | 0 |
| **Total Attempts** | **578** | 458 | 78 | 42 |

**Supplementary Table S2. Types of salvage attempts (analytic cohort only)**

|  |  | **Type of Salvage Attempts** | | | |
| --- | --- | --- | --- | --- | --- |
| **Salvage Attempt** | **Total Number of Salvage Attempts** | **Multi-agent Chemotherapy** | **Novel Agent* ± Chemotherapy** | **Chemotherapy + HSCT** | **Novel Agent ± Chemotherapy + HSCT** |
| First | 221 | 133 | 12 | 69 | 7 |
| Second | 201 | 124 | 8 | 65 | 4 |
| Third | 89 | 53 | 13 | 23 | 0 |
| Fourth – Eighth | 67 | 43 | 14 | 10 | 0 |
| Total (%) | 578 (100%) | 353 (61%) | 47 (8.1%) | 167 (28.9%) | 11 (2%) |
| **Breakdown for salvage attempts 4^th^ – 8^th^** | | | | | |
| Fourth | 31 | 17 | 9 | 5 | 0 |
| Fifth | 21 | 14 | 4 | 3 | 0 |
| Sixth | 11 | 9 | 1 | 1 | 0 |
| Seventh | 3 | 2 | 0 | 1 | 0 |
| Eighth | 1 | 1 | 0 | 0 | 0 |

***Novel agent is defined by agents that were not approved by FDA before 12/31/2013**

**HSCT – hematopoietic stem cell transplant**
